# ‘It makes us sick’: experiences of air pollution among children with asthma and their caregivers in African countries

**DOI:** 10.5588/ijtldopen.24.0659

**Published:** 2025-09-10

**Authors:** Y.A. Kuyinu, O.O. Adeyeye, E. Addo-Yobo, B. Arhin, B. Barratt, B. Chinoko, O. Goodman, M. Kasekete, R. Masekela, E. Mkutumula, R. Mphahlele, B.T. Mmbaga, H.A. Mujuru, S. Muyemayema, R. Nantanda, J.S. Ngocho, L.M. Nkhalamba, S.K. Owusu, B. Said, S. Shaibu, A. Ssemata, I. Ticklay, L.J. Zurba, G. Mosler, J. Grigg

**Affiliations:** ^1^ Department of Community Health & Primary Health Care, Lagos State University College of Medicine, Ikeja, Lagos, Nigeria;; ^2^ Department of Community Health & Primary Health Care, Lagos State University Teaching Hospital, Ikeja, Lagos, Nigeria;; ^3^ Department of Medicine, Lagos State University College of Medicine, Ikeja, Lagos, Nigeria;; ^4^ Department of Medicine, Lagos State University Teaching Hospital, Ikeja, Lagos, Nigeria;; ^5^ Research & Development Unit, Komfo Anokye Teaching Hospital, Ghana;; ^6^ Environmental Research Group, MRC Centre for Environment and Health, Imperial College London, London, UK;; ^7^ The Lung Health Group, Malawi Liverpool Wellcome Programme, Malawi;; ^8^ Department of Child, Adolescent and Women Health, Faculty of Medicine and Health Sciences, University of Zimbabwe;; ^9^ Department of Paediatrics and Child Health, School of Clinical Medicine, College of Health Sciences, University of KwaZulu Natal, South Africa;; ^10^ Department of Paediatrics and Child Health, University of KwaZulu Natal, Durban, South Africa;; ^11^ Paediatric and Child Health Department Kilimanjaro Christian Medical University College, Tanzania;; ^12^ Kilimanjaro Clinical Research Institute, Kilimanjaro Christian Medical Centre, Moshi, Tanzania;; ^13^ Department of Paediatrics and Child Health, University of Zimbabwe, Harare, Zimbabwe;; ^14^ Makerere University Lung Institute, College of Health Sciences, Makerere University, Uganda;; ^15^ Department of Epidemiology and Applied Biostatistics, Kilimanjaro Christian Medical University College, Moshi Tanzania;; ^16^ Department of Child Health, School of Medicine and Dentistry, Kwame Nkrumah University of Science and Technology, Ghana;; ^17^ Department of Child Health, Komfo Anokye Teaching Hospital, Adum-Kumasi, Ghana;; ^18^ Kibong'oto Infectious Diseases Hospital - Sanya Juu Siha, Kilimanjaro, Tanzania;; ^19^ Nelson Mandela Institution of Science and Technology, Arusha, Tanzania;; ^20^ Department of Psychiatry, college of Health Sciences, Makerere University, Uganda;; ^21^ Department of Global Health and Development, London School of Hygiene and Tropical Medicine, UK;; ^22^ Child and Adolescent Health Unit, Department of Primary Health Care Sciences, University of Zimbabwe, Harare, Zimbabwe;; ^23^ Education for Health Africa, Durban, South Africa;; ^24^ Centre for Genomics and Child Health, Blizard Institute, Faculty of Medicine and Dentistry, Queen Mary University of London, London, UK.

**Keywords:** perception of air pollution, experiences among adolescents, mitigation of air pollution, Ghana, Nigeria, Malawi, South Africa, Tanzania, Uganda, Zimbabwe

## Abstract

**BACKGROUND:**

This study was carried out to document experiences and perceptions of air pollution among adolescents with asthma symptoms, and their caregivers.

**METHOD:**

A qualitative study among children with symptoms of asthma (aged 12–16 years) and their caregivers was conducted in seven African countries (Ghana, Nigeria, Malawi, South Africa, Tanzania, Uganda and Zimbabwe). Focus group discussions were used to collect data from children and caregivers and a total of 41 sessions (children 21, caregivers 20) were held. Analysis of the data gave rise to two major themes (perception of air pollution and mitigation against air pollution) from which subthemes were derived.

**RESULTS:**

Participants identified causes of air pollution as cigarette smoking, use of wood for cooking, burning of garbage, generator, presence of industries in residential areas; and that these worsened asthma symptoms. The experiences the children had encountered were at home, while commuting to school and in school. Personal mitigation measures and governmental measures were suggested.

**CONCLUSION:**

It is recommended that governments implement policies to mitigate air pollution and to encourage individuals to adopt personal measures to mitigate its impact.

Air pollution refers to contamination of the air (indoor or outdoor) by any physical, chemical, or biological agent that alters the natural characteristics of the atmosphere.^1^ The introduction of these agents in a certain concentration and duration can cause harm to man, animals or plants. Air quality index (AQI) is used in reporting air quality and it is estimated that 8 out of 10 persons in the world are exposed to an AQI value above 100 (normal air has an AQI of less than 100). The majority of cases occur in low and middle income countries (LMICs) and among those living in urban areas.^2^ This is due to rapid industrial growth, which is made worse by exposure to traffic related air pollutants (TRAP) from heavy vehicles and power generation, and weak or nonexistent environmental protection laws, leading to levels of air pollution that often exceed emission standards set by the WHO.^3,4^ Air pollution is recognized as a major cause of morbidity and mortality.^5^ Exposure to air pollutants has potentially harmful effects, such as increased risks of lower respiratory tract infections, asthma, chronic obstructive pulmonary disease (COPD), psychological symptoms, increased school absenteeism for children, visual information processing speed, and stress – as well as long-term effects that affect susceptibility to disease later in life.^5,6^ Children are especially vulnerable due to their developing bodies^7,8^ and evidence shows that the lungs of children exposed to higher levels of air pollution have lower lung volume than those in cleaner areas.^9^ Lower lung function tracks throughout the lifespan with life-long impact on health and quality of life.^10^ Air pollution imposes additional challenges on the respiratory health of children with asthma, making them especially sensitive to repeated pollution exposure.^11^ Apart from increasing the burden of asthma, it may result in increased use of asthma medications, poor asthma control, and increase in asthma related health care utilization.^12–15^

Although studies on personal exposure and perception of adverse effects of air pollution are becoming common in high income countries (HIC) especially in Europe, no large-scale studies have been carried out in Africa, which has significantly different pollutant sources and patterns of exposure. There is therefore insufficient evidence for policy and intervention. Here, we have documented the experiences and perception of air pollution among adolescents with asthma symptoms, and their caregivers, to help us understand the health of Africa’s young population.

## METHODS

This was a qualitative study using a cohort of children with asthma aged 12–16 years and their caregivers in seven African cities: Ghana (Kumasi), Malawi (Blantyre), Nigeria (Lagos), South Africa (Durban), Tanzania (Moshi), Uganda (Kampala) and Zimbabwe (Harare). This study is a component of the National Institute for Health and Care Research (NIHR) funded Global Health Research Group ‘Children’s air pollution profiles in Africa’ (CAPPA) study, led by Queen Mary University of London, UK. The CAPPA study included personal air pollution monitoring,^16^ and peak flow testing of participants, in addition to a qualitative element presented in this paper. The present study was preceded by an earlier study (titled Achieving Control of Asthma in Children in Africa (ACACIA),^17^ which included six of the sites and enabled researchers to have prior interaction with the study participants. The site in Tanzania was added to the group for the air pollution element of the study.

### Participant selection and data collection

The original six study sites selected children aged 12–16 years with symptoms of asthma that were involved in the ACACIA study. The selection was done in collaboration with the schools, based on what was practically feasible considering COVID-19 restrictions, whilst capturing sufficient diversity in school sites, home environment and transport habits. The newly added site in Tanzania used ISAAC (International Study of Asthma and Allergies in Childhood)-study questions, following the ACACIA protocol to recruit children with asthma symptoms.^17^ Potential participants and their caregivers (who are family members) were invited to participate.

Data were obtained from participants who met the research teams in rooms at schools and halls within hospitals located in urban centres of the country sites between August 2021 and July, 2022. Focus Group Discussions (FGDs) using a FGD guidelines (that were agreed upon by all participating countries) was used to obtain information from the children about perception and experiences with air pollution. A total of 21 FGD sessions (among children) and 20 FGD sessions (among caregivers) were carried out in 7 countries (Table 1) with duration lasting 45–70 minutes. Each country had participants, 2 researchers and 1 note taker constituting a discussion group; and an audio recorder was used to ensure adequate data collection. The participants in each group comprised 5–10 persons and sessions were conducted in English (except for participants in Tanzania and Zimbabwe where sessions were conducted in Swahili and Shona respectively and subsequently translated to English by researchers in both countries who are native speakers of both languages).

**Table 1.** Distribution of participants in Focus Group Discussions (FGDs) by country.

Country	Number of FGDs (children)	Number of FGDs (caregivers)	Total Number of FGDs	Number of participants (children)	Number of participants (caregivers)	Total Number of participants
Nigeria (NG)	3	3	6	24	24	48
Ghana (GH)	3	3	6	21	18	39
Uganda (UG)	2	3	5	15	22	37
Zimbabwe (ZM)	3	3	6	14	20	34
Tanzania (TZ)	3	3	6	21	21	42
Malawi (MW)	3	1	4	12	7	19
South Africa (SA)	4	4	8	21	21	42
Total	21	20	41	128	133	261

Audio recordings were transcribed by the note-takers and researchers in individual study sites; and inaudible segments were filled in with the notes taken. Analysis of data was done using the step-by-step guide on thematic analysis.^18^ A coding team was formed (comprising a representative researcher from each country team) to independently develop a codebook for the study and the codebook was agreed upon by all countries. Codes were subsequently generated and coding was done by all involved researchers using the software, NVivo 1.6 (QSR International [Lumivero], Massachusetts, USA). Investigator triangulation was utilized to ensure adequate data capture and interpretation. Based on data obtained, several themes were identified and two major themes were agreed upon. Subthemes also emerged based on relationship to the major theme (Table 2); some subthemes were merged as the participants essentially expressed the same ideas. The first theme, perception of air pollution, had six subthemes and the second, mitigation measures, addressed two subthemes (personal measures and governmental measures) – see Table 2. Data was reported in accordance with the consolidated criteria for reporting qualitative research (COREQ).^19^

**Table 2.** Major themes and subthemes.

Major theme	Subthemes
Perception of air pollution	Definition
	Causes
	Fuels that worsen air pollution
	Role of cooking and other domestic activities on air pollution
	Concerns & effects
	Experiences
Mitigation measures	Government measures
	Personal measures

### Ethics statement

This study was reviewed by the Queen Mary University of London Ethics of Research Committee in the UK. Each collaborating African centre received ethical approval for the study locally: Ghana (GH) CHRPE/AP /030/22; Malawi (MW) P.04/21/3301; Nigeria (NG) LREC/06/10/1484; South Africa (SA) BREC/00002420/2021; Tanzania (TZ) NIMR/HQ/R.8a/vol.IX/3547; Uganda (UG) MHREC 2056; Zimbabwe (ZM) MRCZ/A/2415. All study participants consented to the study, and anonymity was maintained by using participant identifiers instead of names.

## RESULTS

A total of 128 children and 133 caregivers were involved in the study (Table 1). The children and caregivers had an idea of the definition of air pollution as expressed in various ways to communicate its concept (Table 3). In expressing the causes of air pollution, a number of the participants mentioned these causes while talking about the definition of air pollution. Some participants were also able to identify sources of air pollution as dusts, perfumes, smoke from cigarettes, marijuana, stoves, cars, generators, factories, and other smoke producing activities. Participants used expressions such as:

‘I think excessive dust is a cause of air pollution.’ - child GH

**Table 3.** Perception of air pollution.

Sub theme	Quotes from participants
Definition of air pollution	‘Is a harmful substance that occur in the air.’– child GH ‘air pollution is the contamination of our environment, indoor and outdoor in that outdoor, our environment can be polluted by the factories.’– caregiver UG
Causes	‘Air pollution is when the air is contaminated for example smoke from cars, smoke from industries it is harmful for the body.’– child NG ‘It is dirty air and this dirt is caused by different things for example smoke, exhaust fumes, gases.’– caregiver ZM
Fuel that worsen air pollution	‘Using firewood can contaminate the air.’– caregiver NG
Role of cooking and other domestic activities on air pollution	‘Deep frying of palm oil will add to air pollution.’– caregiver NG ‘When you light the charcoal stove sometimes you use polythene…there is air pollution.’ – caregiver UG ‘if you are sweeping, that causes dust to rise. So you can breathe that in and it can cause you to start breathing abnormally.’–child MW ‘use of insecticides at home. We are discouraged to inhale it as can make us cough.’– child NG ‘for me I have experienced it on my child. Sometimes the siblings can bring perfume and spray themselves and you hear her affected and yet the perfume has a scent.’– caregiver UG
Concerns and effects of air pollution	‘It makes us sick…’–child SA ‘it makes one feel dizzy.’– child NG ‘Yeah, because sometimes it triggers serious coughing and you fail to breathe at night and because you met these smokes during the day, it can trigger your asthma and you fail to breathe. So it can really make a person weak.’– child MW ‘staying in places that have a lot of smoke and perfume smells are also not good. It causes problems to people with asthma.’– child MW ‘…because if the weather is dirty it can cause damage to the lungs other diseases can occur like the chest stinging.’– child TZ ‘Also, air pollution, especially from industry, can cause global warming and death. Also for those with asthma it can cause more side effects.’– child TZ ‘We are all concerned about air pollution because it is major cause of respiratory diseases such as asthma in the world, especially in Africa.’– caregiver GH ‘Because it makes our children’s chests congested, and it makes them have problems with their chests.’– caregiver SA. ‘It could be because we are breathing in so it’s causing headaches and migraines and tiredness. It doesn’t just affect you that you can’t breathe, like with asthma and all, but it’s also other things too. When you suffer from brain fog, it makes you tired. It affects your daily life. You can phone and say, ‘I can’t make it today,’ because of the way you are feeling. You are fatigued. You can’t get out of bed because it’s affecting other areas besides just feeling like I can’t breathe. You are tired from other things’– caregiver SA

‘Sometimes we take a charcoal stove and put it inside the house yet it produces smoke that may end up affecting those who are asthmatic and if an individual is putting up in such kind of a room he or she may end up suffocating.’ - child MW

On cooking fuel, the participants identified charcoal, firewood and kerosene stove as fuel that made air pollution worse. The role of cooking and other domestic activities on air pollution was explored and caregivers were responsive in this regard (Table 3). Concerns about air pollution were expressed in terms of its effects on the human health: the feelings one gets from it, respiratory diseases and shortened life span. Caregivers’ major concerns were for their children. The children were more expressive regarding concerns about air pollution (Table 3) as one of the children participants group had expressed:

‘It makes us sick…*’ -* child SA.

A caregiver expressed the concern of the effect of air pollution on buildings and the homes:

‘Living close to the refinery, I know from working in the refinery that the refinery actually pays for curtains, windows and roofing of nearby residences to get washed. If you were directly across the road, it’s one road that separates them. Our windows get pitch black. We have to wash in a week. Within three days your windows are dirty. If you rub the windows like this, it’s thick black. I also think it’s the particles from the refinery.’ - caregiver SA

A few of the children and the caregivers did not have a good understanding of the effect of air pollution:

‘I'm concerned because in our environment where there's air pollution it can bring about many diseases. cholera, typhoid can be caused by air pollution’ *-* caregiver NG

All participants agreed that a relationship existed between asthma and air pollution, which was described as asthma condition being worsened by air pollution. Based on this, participants recounted various experiences they had with air pollution (Table 4). The theme mitigation measures focused on personal measures and governmental measures to reduce air pollution. Exploring personal measures, some participants suggested actions that could be carried out as use of face masks, avoiding smoke and insecticide in addition to other identified measures individuals can put in place to prevent air pollution (Table 5). Some participants mentioned steps they were currently taking.

**Table 4.** Experiences of air pollution.

Sub theme	Quotes from participants
Experiences of air pollution	‘Burning of litter in the morning on the dumpsite close to my home so I pass through there when going to school which makes me start to cough. Of which it’s the shortest route’– child MW ‘I went to church, and I was seated with a friend, and he used a strong scented spray I could not breathe properly, so I quickly came out from the place for fresh air’. child GH. ‘They had just painted our house and then they were also cooking inside the house with a charcoal burner. So as I was sleeping in my room, I started having difficulties with breathing until I was unconscious. When they realised it, they took me outside for some fresh air that was when I was better.’ child MW ‘we have crazy kids at our school. So yes, they were smoking. So each time I would smell that strong smell, I would start having difficulties in breathing or sometimes just sneeze’– child MW ‘before coming to school while walking along the road, some people smoking cigarettes makes me to start coughing.’– child NG ‘Many times, when buses came past and the smoke— exhaust fumes, it actually chokes me.’– child SA ‘I used to stay with my uncle who was a chain smoker and I used to be sick all the time and he would smoke inside the house. But he used to say that I am a weakling and should man up. It all stopped when my mother took me back home.’– child ZM ‘In 2019, my cousin came to spend holiday and I was not aware that she's asthmatic suddenly she was gasping after frying in the kitchen so I shouted for help and some drugs were administered to her. She also said her father is asthmatic’– caregiver NG ‘There was a day I went with my daughter to Kyaggwe, the roads were so dusty. When we came back the following day, she got a very bad attack’– caregiver UG

**Table 5.** Mitigation measures to prevent air pollution

Sub theme	Quotes from participants
Personal measures	‘Use Nose mask, avoid using a smoky bus and educate children on air pollution.’– caregiver NG ‘By staying away from dusty roads.’– Caregiver NG ‘Encourage regular servicing of the generator to reduce smoke.’– Caregiver NG ‘Reduction in the use of insecticides and coils’.– caregiver GH ‘Avoid sleeping in insecticide spray immediately after spraying’.–Child GH ‘Tell people to stop smoking and to stop throwing papers into the river’– child SA ‘I will change my route back home to avoid fumes from trucks and public transport. My house is a walking distance from my school, so I used to walk by the roadside to school and the road is too busy, so I will change my route’– child GH ‘Yes I just want to say that we need to be avoiding all things that can trigger our asthma. We might think these things are good but at night as we sleep, some caregivers sometimes go away for overnight prayers or work and you can die alone because you can’t breathe. This disease is dangerous, so we need to be avoiding those things’– child MW
Governmental measures	‘…… introduction of electric cars into the society’– caregiver NG ‘Measures to ban cars which release a lot of exhaust fumes’– caregiver ZM ‘Public smoking should be an offense and there should be no smoking zones’– caregiver ZM ‘Public enlightenment on the need to prevent indiscriminate burning of refuse’– caregiver NG ‘Strict penalties for offenders like those people who burn rubbish’– caregiver ZM ‘Maybe you can help us petition the city council so that they can collect refuse so that people will not resort to burning rubbish’– caregiver ZM ‘I want government to implement a law to guide companies with exhaust’– caregiver NG ‘There should be regulations that bind industries on air pollution and there should be air pollution monitors at the industrial sites’– caregiver ZM ‘Industries should not be situated around residential area. We do not have regular power supply, so if the government van provide steady power supply it will go a long way’– caregiver NG

‘The action I took was not to let my children play in the dust. In the dusty season, I try to keep watering, at least the dust is not too deep. Unfortunately, my children and I have been affected by asthma; so if it happens inside, there is a dusty situation we are already trapped. So I always try to keep the environment humid’ - caregiver TZ

‘We cannot avoid burning rubbish, but it requires you check for the direction of the wind. So, if you are burning and you stand opposite to where the wind is moving, or cooking using firewood, you don’t sit there until the food is ready’ - caregiver UG

On the subtheme of government measures, the caregiver group participants contributed to the discussion, whereas the child group participants made no contribution. The measures suggested by participants in the caregiver group were in terms of policies and penalties for violation (Table 5).

## DISCUSSION

The participants across all countries had an idea of the definition of air pollution. This finding is similar to findings among students in a Greek study^20^ and adults^3^ who had used the same terminologies as ‘dirt’ and ‘contamination’. There was confusion between air pollution and environmental pollution as some participants kept referring to littering with pieces of paper and bad smell. This confusion should be targeted and given attention in air pollution interventions. However, the participants were able to rightly identify sources of air pollution. The issue of burning of garbage was recurring across all participating countries, which is a pointer to specific pollution issues and economic development of the African sites. The role of cooking in producing indoor air pollution is attributed to the cooking methods in Africa, such as bleaching of palm oil and frying. Other domestic activities such as sweeping (which produces dust) and smoking of cigarettes were identified. Rightly identifying these methods of cooking and activities will help to provide personal measures to mitigate indoor air pollution. The cooking fuels identified to produce air pollution were common across countries and this reflected in the experiences shared by various participants. These sources of fuel are well reported by other studies.^3,21^

Across countries, it was commonly expressed that air pollution worsened asthma and this was well described by the experiences of the students and their caregivers. A number of these students associated their experiences with their commuting to school (walking or on a bus). Given that there are limited route options and public transport availability in the African setting, this implies that these students may not escape from trigger factors whether indoor or outdoor. This would impact on the frequency of asthma episodes as a previous study^15^ linked traffic related air pollution to increased asthma episodes and more hospitalizations. This may corroborate the concerns of the children and their caregivers as it would impact on the unease it posed, medical expense and school absenteeism.^22,23^

Some of the experiences documented in this study are alarming such as ‘a child having difficulties with breathing until he/she was unconscious’ and this should raise concern among communities, leaders, education sectors, media and governments. Media advocacy can be used to highlight issues around air pollution to inform individuals, communities and policies, as an earlier study in Africa^24^ had highlighted that the causes of air pollution were the least reported aspect of air pollution.

To mitigate air pollution effects, it is important for people to identify and understand the sources and impact of air pollution. This is particularly true for people at risk of adverse effects from air pollution, such as asthma. An earlier study ^16^ (among the same participants) identified the use of biomass fuel for cooking, non-paved school floors, indoor smoking and commuting as a major exposure to air pollution for these children (based on particulate matter [PM] measurements and monitoring) and these air pollution sources were also identified during the FGD. Identified changes as suggested by the earlier study will be helpful at the individual and community level.

Participants suggested measures to reduce air pollution production and exposure which have been documented as strategies likely to reduce pollution by other studies.^4,25,26^ The suggested personal measures are effective for indoor pollution and will become effective as an outdoor air pollution control if carried out collectively. For instance, garbage burning was a recurrent code across countries and if one individual stops this activity, high outdoor air pollution will persist if neighbours do not desist from burning. This emphasizes how governments can help mitigate the menace of air pollution by putting in place mechanisms to manage waste thereby preventing burning by individuals; as many people feel ‘helpless’ as expressed in an earlier study.^3^

Policy as suggested in this study may be good for control of air pollution, but implementation of these policies are more important. Other studies^27,28^ recommended optimization of vehicle filtration (keeping windows closed and set to maintain internal air circulation) in conditions of high air pollution while in transit. However, this may not be practical because public transport does not have functional air conditioning. Some studies^29–31^ have recommended substitution of biomass cooking fuel for gas and electric fuels, but the sustainability of this in these countries remains a question given the socioeconomic status of most people who may not be able to afford to upgrade to low emission cookers, as well as gaps in the electricity or gas infrastructure. Air pollution monitoring as practiced in some countries^32,33^ may be considered by governments in Africa. Personal measures to mitigate air pollution (such as desisting from burning garbage, staying away from dusty roads, avoiding cigarette smoking in public places, use of cleaner cooking fuels) are practicable for our settings. If promoted and embraced by every individual, these will have a positive, collective effect. With collective personal measures in place, additional measures put in place by the government will help to combat air pollution.

A strength of this study is its qualitative nature, which provided the opportunity to explore these important air pollution issues. However, a limitation is that the participants might not be representative of the wider public. They were selected based on asthma symptoms and have been part of a prior study on air pollution; they may therefore have a better understanding and more specific concerns compared to the wider public. In our study, children with asthma and their caregivers had an idea of air pollution, its sources in their environment and the exacerbating effect air pollution has on asthma, as attested to by the experiences shared. The concerns expressed by the participants suggests that there might be motivation to participate in or advocate for change.

It is recommended that personal measures (such as preventing burning of garbage, cooking with coal indoors) be adopted. The media can also help to raise awareness of the damaging effects of air pollution and governments should make and implement policy to control such activities as smoking and garbage disposal –and empower individuals socio-economically to afford healthier cooking alternatives. Provision of a stable electricity supply will increase the use of electric cookers and bring an end to the use of generators. We also recommend that governments use air pollution monitoring and strictly maintain a separation between residential and industrial areas to reduce exposure to air pollution.
